# Neurotrophin levels at admission did not change significantly upon alcohol deprivation and were positively correlated with the BMI and LDL levels

**DOI:** 10.1186/2049-9256-1-20

**Published:** 2013-12-02

**Authors:** Aurel Popa-Wagner, Karolina Furczyk, Joerg Richter, Gisela Irmisch, Johannes Thome

**Affiliations:** Clinic for Psychiatry and Psychotheraphy, University of Medicine Rostock, Rostock, Germany; Norway Centre for Child and Adolescent Mental Health Eastern and Southern Norway, Oslo, 0405 Norway

**Keywords:** Alcoholism, Brain-derived neurotrophic factor, Neurotrophin-derived factor 3, Detoxification, Lipoproteins

## Abstract

**Background:**

The neurotrophins brain-derived neurotrophic factor (BDNF) and neurotrophic factor 3 (NT3) could play a role in addictive behavior. Interactions between BDNF and dopamine transmission influence the alcohol intake. It has been hypothesized that extensive alcohol consumption leads to diminished circulating BDNF levels and impaired BDNF-mediated protective mechanisms. What is more, alcohol dependency causes changes in lipid metabolism which in turn may influence the neurotrophin system.

**Methods:**

In this study, we tested the hypothesis that alcohol withdrawal increases the serum levels of BDNF in alcoholic patients and investigated correlations between serum BDNF and NT3 and alcohol in breath as well as with the body-mass-index (BMI), lipoprotein profiles and lifestyle factors in 110 male in-patients diagnosed with alcohol addiction on the first day after admission and at discharge.

**Results:**

The intoxication level (alcohol in breath at admission) was significantly correlated with liver enzymes and BDNF concentrations (R = .28; p = .004). Patients with positive breath-alcohol test at admission had about 9 times higher NT3 levels and higher liver enzyme concentration levels than nonintoxicated subjects. Alcohol intoxicated patients with pathological aspartate aminase (ASAT) levels had even higher NT3 level (F = 5.41; p = .022). The concentration of NT3 was positively associated with the (BMI) (admission R = .36; p = .004; discharge R = .33; p = .001), and the obese patients had 3 to 5 times higher NT3 concentration than the others. Low-density lipoprotein (LDL) concentration levels were found to positively correlate with NT3 concentration levels (admission R = .025; p = .015 discharge R = .24; p = .23).

**Conclusion:**

Other than expected, the levels of NT3 and to a lesser extent BDNF levels, were found to be significantly increased in acute alcohol abuse. Alcohol deprivation did not significantly change the serum neurotrophin levels at admission. NT3 levels were positively correlated with the BMI and LDL levels. Because of expected difference between genders, we recommend investigating these correlations further in patients of both genders.

## Background

BDNF is presumably involved in neuroadaption to addictive behaviour. Interactions between BDNF and the dopamine transmission may influence the alcohol ingestion [1, 2]. Extensive alcohol consumption dysregulates the BDNF-mediated schelter mechanisms resulting in further increase of alcohol ingestion. The BDNF mRNA expression in the Striatum Dorsalis depends on the duration of alcohol abuse. After six weeks of alcohol consumption the BDNF level in the cortex was shown to decrease, and even after a two-weeks abstinence it did not normalize. Other studies revealed a region-specific upregulation of BDNF after alcohol consumption [3]. Huang and colleagues [4] found that BDNF serum concentration in alcoholic patients has normalized after just one-week withdrawal. However, permanent changes in BDNF signalling could influence the addiction [5]. A decreased BDNF-expression or attenuated signal transduction through the BDNF-receptors in the presence of alcohol could also contribute to alcohol-induced neurodegeneration. Therefore, it has been speculated, that the administration of BDNF could protect against alcohol abuse. Additionally, a significant correlation between serum-BDNF and the number of abstinence days was also described. As a result, BDNF could be used as a biomarker for abstinence in addictive patients. In subjects with a family history of alcohol abuse over several generations, an interaction was detected between allele variants of BDNF genes associated with increased volumes of brain gray substance and the addictive behaviour [6]. Furthermore, the comorbid psychiatric diseases and their influence on developing alcohol dependency could be of interest [3].

BDNF also plays a role in sleep disturbances associated with alcohol abuse. Since BDNF plays an important role in the glutamate/light/induced phase-change [7], alcohol-associated sleep disturbances that are determined by glutamate-induced phase shifts of the circadian rhythm can be attenuated by increasing the BDNF levels.

But not only psychical influences and the severity or form of alcohol abuse may be important for the neurotrophin expression, but also the metabolic status: for example, BDNF concentrations were found to be influenced by glucose intolerance in alcoholic patients. After alcohol abstinence, BDNF levels were significantly increased in pre-diabetes mellitus patients, but not in patients with a normal glucose tolerance or in diabetic patients [8].

Alcohol misuse has serious effects on the liver metabolism and the lipoprotein levels in serum [9]. Correlations between lipoproteins and neurotrophins were also found in mice treated with cholesterol (Chol) reducing drugs like statins which increased the hippocampal BDNF levels with a concomitanly antidepressant-like effect [10]. In patients with angina pectoris, plasma BDNF was inversely associated with low-density lipoprotein (LDL) cholesterol levels, and positively associated with high-density lipoprotein (HDL) cholesterol levels [11]. High dietary cholesterol intake elevated BDNF mRNA expression in mice [12]. In young men, serum BDNF was positively correlated with body mass index and total cholesterol [13]. Since the benefits of the omega-3 polyunsaturated fatty acids (omega-3 PUFA) found in fish oils may be attributed to a number of distinct biological effects on lipoprotein metabolism like the reduction of triglycerides and the possible reduction of pro-atherogenic small dense LDL particles (sdLDL) [14], it is possible that the dietary intake of fish also affects the neurotrophin levels.

We are not aware of publications about the influence of ethanol on NT3 concentrations in serum. However, in one study it was reported that the exposition to ethanol did not influence the NT3 mRNA expression in the brains of rat pups [15]. In another study done on cultured cortical neurons, NT3 treatment did not attenuate the toxic effect of ethanol [16]. Others found elevated NT3 levels in different brain areas of juvenile rats prenatally exposed to alcohol [17].

In this study, we investigated correlations between serum BDNF and NT3 with alcohol in breath at admission as well as body-mass-index, lipoprotein profile and lifestyle factors in 110 male in-patients diagnosed with alcohol addiction on the first day after admission and at discharge. After detoxification, we expected an increase in the serum concentration of BDNF and NT3 [18, 19].

## Methods

### Ethics

This study was approved by the Institutional Review Boards of the University of Medicine the Rostock. All subjects provided formal consent before being examined and were compensated up to 50 EURO.

### Subjects

The patients were volunteers for alcohol detoxification treatment in the psychiatric University Hospital. A total of 110 alcoholic male patients were enrolled with mean age of 44.5 ± 9.8 years and BMI 24.5 ± 3.8. BMI was calculated according to the formula Weight (kg)/Height (m)^2^. Alcohol addiction was judged by symptoms of alcohol withdrawal severe enough to require pharmacologic treatment. Exclusion criteria included a history of other psychiatric disorders than alcohol abuse, neurological illness, current or unstable medical illnesses or disability. Individuals on any kind of ongoing vitamin substitution were also excluded from the study. All patients were free of psychiatric medication at the time of neuropsychological testing and blood parameter determination. Demographic characteristics of the participants are shown in Table 1.

**Table 1:** **Case demographics**

	N	Mean ± SD
Age [years]	110	44.76 ± 9.55
Body-mass-index	109	24.46 ± 3.75
Days between assessments	106	10.83 ± 2.44
Daily alcohol consumption	104	30 U ± 10
Years of addiction	104	9.30 ± 6.64
% Frequency of fish meals/week		
Never	14	12.8
<2 weekly	83	76.1
≥2 weekly	12	11.0
Smoker	100	91.7

#### Alcohol consumption and biochemical analysis

The average alcohol consumption (1 alcohol unit = 20 g alcohol/d) and the duration of alcohol abuse were assessed (Table 1). For fatty acid and liver enzymes Gamma-glutamyltransferase (GGT) and Alanine-Aminotransferase (ALAT) and Aspartate-Aminotransferase (ASAT) analysis a blood sample was drawn from the alcoholic patients at the morning after hospital admission and after the detoxification, at the day of discharge from the hospital. Additionally, serum levels of the brain-derived neurotrophic factor (BDNF) and neurotrophic factor-3 (NT3) were also measured. As a criterion of the severity of alcohol disease, we recorded the liver enzymes ASAT, ALAT and GGT and calculated their influence on the neurotrophin and lipoprotein levels.

#### Analytical methods

BDNF and NT3 were analysed using ELISA test kits from Ray Bio®. The level of total cholesterol (Chol) and the HDL and LDL lipoproteins were analysed using a kit from AXONLAB ® run on the Cobas Fara Analyzer (Roche, Switzerland). Liver enzymes ASAT, ALAT and GGT were measured as previously described [20].

#### Statistical methods

Mean scores and standard deviation were reported from continuous variables; absolute and relative frequencies were described for categorical data. Paired-sample *t*-test was used to test for differences between the two assessments of the biochemical analysed concentrations. Spearman rank correlations were applied to test for relation between continuous variables, because most of them were found not to be normally distributed (by Kolmogorov-Smirnov-test). To test for relation between categorical and continuous variables, one-way analysis of variance (ANOVA) was applied. The significance level for all tests was set at p < 0.05. Because of the explorative nature of our study, we did not correct the level of significance of our findings for multiple testing. Data were analysed using SPSS version 17 (SPSS Inc., Chicago, IL, USA).

## Results

### Case demographics

The patients were 44.76 ± 9.55 years old (ranging from 18 to 67 years) and had a history of alcohol addiction of 9.30 ± 6.64 years (ranging from 1 to 31 years). 32 patients showed up at admission with negative breath-alcohol test (29.1%). The BMI of the participants was 24.46 ± 3.75 (ranging from 16 to 35). The time difference between the both assessments was, on average, 10.83 ± 2.44 days (ranging from 4 to 16 days). The vast majority of the alcohol dependent patients reported to eat fish two times a week (76.1%); none of them was a vegetarian; 91.7% were smokers (Table 1).

The frequency of pathologically high concentration of various liver enzymes was as follows: ASAT – n = 62 (56.4 ALAT – n = 54 (49.1%), and GGT – n = 81 (73.4%). Only seven patients (6.4%) could be classified as suffering from a mild dependence on alcohol based on the criterion of having no pathologically elevated liver enzyme and showing-up at admission with negative breath-alcohol test versus either showing at least one pathologically elevated liver enzyme or showing positive breath alcohol levels at the time of admission. As might have been expected, these two groups did not differ in any of the analysed lipoproteins, neurotrophins or liver enzymes concentration in serum because of the very small number of ‘mildly dependent’ patients (Table 2).

**Table 2:** **Comparison of parameter concentration between admission and discharge (paired-sample** ***t*** **-test)**

	N	Admission	Discharge	Reference	T	Df	P
BDNF [ng/ml]	104	183.97 ± 92.07	173.38 ± 96.07		1.16	99	0.249
NT3 [pg/ml]	93	262.51 ± 695.91	263.67 ± 674.51		−0.24	87	0.808
HDL [mmol/L]	107	2.35 ± 0.99	1.69 ± 0.52	1.1-2.3 mmol/L	7.24	106	< 0.001
LDL [mmol/L]	110	3.29 ± 1.22	3.41 ± 1.15	<3 mmol/L	−0.90	107	0.369
Cholesterol, total	109	6.60 ± 1.65	5.83 ± 1.59	<6 mmol/L	4.72	106	< 0.001
ASAT [U/L]	110	93.55 ± 91.03		<50 U/L			
ALAT [U/L]	110	72.13 ± 63.16		<50 U/L			
GGT [U/L]	110	284.51 ± 444.48		<60 U/L			
Breath alcohol	110	1.20 ± 1.11					
[‰]							

### Socio-demographic and illness-related variables in relation to neurotrophins, lipoproteins, and liver enzymes

The age of the subjects was significantly correlated with ASAT and GGT concentration in the blood and showed a tendency for an association with BDNF at both assessments. The alcohol breath concentration was significantly correlated with ASAT and ALAT levels in blood and with BDNF concentration at admission. There was no significant correlation between age, alcohol in breath and lipoproteins or NT3 concentration in blood at both assessments (Table 3).

**Table 3:** **Correlations of age and breath alcohol with selected neurotrophin and liver enzyme concentrations**

	Age [years]	Breath alcohol [‰]
BDNF admission	−0.17 (0.095)	0.28 (0.004)
BDNF discharge	−0.18 (0.077)	0.03 (0.737)
ASAT	0.23 (0.016)	0.43 (<0.001)
ALAT	0.07 (0.495)	0.28 (0.003)
GGT	0.27 (0.004)	0.06 (0.549)

The BMI was significantly associated with the NT3 concentration at both assessments (admission: R = 0.36; p = 0.004); discharge: R = 0.33; p = 0.001) with the group of obese patients (n = 10; BMI > 30) having, on average, three to five times higher NT3 concentration than the others; as well as with the HDL concentration at admission (admission: R = −0.33; p <0.001). It did not, however, correlate with the HDL concentration at discharge: R = −0.14; p = 0.141) or with any other liver enzyme or lipoprotein concentration.

The longer the period of inpatient treatment, the higher the BDNF concentration at admission (R = 0.20; p = 0.042); the lower the NT3 concentration (admission: R = −0.45; p < 0.001; discharge: R = −0.46; p < 0.001); and the lower the breath alcohol concentration (R = −0.22; p = 0.021). It is worth mentioning that the period of inpatient treatment depends on the one hand on the patient’s motivation to undergo the therapy and to take part in the psychotherapeutic program, on the other hand, on the severity of the withdrawal syndrom. The patients with the lower breath alcohol concentration on admission could have been stronger motivated for the therapy, since they already had reduced their alcohol intake prior to admission, therefore they completed the full-length therapy program. Still, the same patients could also experience more severe withdrawal symptoms because of their attempt at withdrawal without supporting medication, also resulting in a longer inpatient treatment. The patients with negative breath-alcohol test at admission had about nine times lower NT3 concentration both at admission (F = 3.75; p = 0.056) and at discharge (F = 4.64; p = 0.034; as well as lower ASAT (F = 16.03; p < 0.001) and ALAT concentration (F = 7.01; P = 0.009).

As expected, the more alcohol units the patients reported to drink per day, the higher was the HDL concentration at discharge (R = 0.27; p = 0.006). Likewise the HDL concentration at discharge correlated with the duration of treatment (R = 0.24; p = 0.015) and the number of years of alcohol dependence (R = 0.21; p = 0.036). Smokers had a tendency of lower NT3 concentration compared to non-smokers at both assessments (admission: F = 3.84; p = 0.53; discharge: F = 3.60; p = 0.061). Smoking was not related to any patient-related background variables or to other neurotrophins and lipoproteins, or liver enzyme concentrations in our investigation. The presence of fish in the diet was unrelated to all assessed parameters and variables (Table 2).

### Liver enzymes, neurotrophins and lipoproteins

The higher the LDL concentration in blood serum was, the higher was the NT3 concentration at admission (R = 0.25; p = 0.015) and at discharge (R = 0.24; p = 0.023). There was no further substantial association between any lipoproteins and liver enzymes concentration or BDNF concentration in the patients. However, the patients with the pathologically high ASAT concentration showed higher NT3 concentration at admission (F = 5.41; p = 0.022) than those with the normal ASAT concentration. This was the only difference in the lipoproteins and neurotrophin concentrations found between the patients with one pathologically elevated liver enzyme and those with normal enzyme concentrations (Table 2).

### Changes in lipoproteins and neurotrophin levels between admission and discharge

There was no significant difference in the neurothropin concentrations at admission and at discharge. The HDL and the total cholesterol concentration were found significantly reduced at discharge as compared to those at admission (Table 2). Seven percent of the variance in the NT3 concentration at discharge could be explained by the variance in the lipoproteins and liver enzymes in a multiple regression with HDL concentration being the only significant variable in the regression equation (standardised Beta = −0.25; t = −2.41; p = 0.018); the variation in BDNF concentration however could not be caused by the same mechanism. The regression models with concentration of the various lipoproteins at discharge as dependent variables and the neurotrophins combined with the liver enzymes from admission as independent variables were all not significant.

## Discussion

It has been reported that in alcohol dependent subjects, serum BDNF-concentration recovers after alcohol deprivation [18, 19]. In our study done on 110 alcohol-intoxicated patients we could not confirm this finding. On the contrary we found that alcohol deprivation did not significantly change the elevated serum neurotrophin levels at admission. In this study, we show in a representative number of subjects, significant correlations between the NT3 concentrations and the BMI index, LDL levels and liver enzyme concentrations at admission. This points to a possible biological association between the neurotrophins and lipoproteins concentration levels in alcoholic patients, which both could be clinically useful biochemical markers in addition to usual laboratory parameters.

Only few and inconsistent results on BDNF, NT3 and lipoprotein concentrations in alcohol dependent patients have been published by now. Zanardini et al. reported a significant reduction in BDNF serum levels in alcohol-dependent patients as compared to healthy subjects [21]. Also binge drinking results in attenuated BDNF levels in humans [22]. In vitro, long-term chronic ethanol exposure results in reduced BDNF signalling in human neuroblastoma cells [23]. Similar results were described in animal models, with reduced hippocampal neurotrophin content [24] and reduced BDNF gene expression [25] in rats after long-term chronic ethanol consumption. Even a short-term exposure to ethanol resulted in a decreased BDNF mRNA expression in the mouse cerebellum [26]. Additionally, ethanol was found to block the BDNF enhancement of postsynaptic NMDA receptors [27] in mice. Other animal study showed that ethanol neurotoxicity may be ameliorated by supplementing with neurotrophic factors [28].

But there are other animal studies relating a quite opposite view on the interaction between ethanol and neurotrophins: Bruns & Miller [29] reported that ethanol increased the number of NGF- and BDNF-expressing neurons and the neurotrophin content per somata in the cerebral cortex of the adult rat. However, after an episodic exposure to ethanol the BDNF content increased and the NT3 levels decreased in the parietal cortex while in the hippocampus the amounts of both neurotrophins increased. Neurotrophin content in the rat basal forebrain was differentially affected by episodic exposure to ethanol: NGF and NT-3 content in the basal forebrain was reduced and NGF and BDNF content in the septal nuclei was increased [30].

Prenatal alcohol exposure in rats increased BDNF expression in the frontal cortex, while environmental enrichment resulted, on the contrary, in lowered BDNF [17]. In mice, the injections of BDNF and NT3 could modulate neuroadaptation to ethanol, potentially influencing the function of postmitotic neurons in the adult brain [31].

It is known that BDNF plays a role in energy metabolism and body weight regulation in humans [32]. Cholesterol and lipoproteins have important functions as structural components of cellular membranes (including neurons) and as precursors of a variety of hormones. A number of studies revealed the association between the plasma [11] and serum [13] BDNF levels and the lipoproteins. Therefore, dietary lipids may impact different pathological processes, possibly in connection with neurotrophic factors.

Weak points of this study are the lacking of a comparison group (ie, nonalcoholic subjects) and the limitation to male subjects only. Another limitation of the study is that 90% of the recruited patients were smokers. There are several studies relating neurotrophins expression in endothelial cells in the airways with smoking [33]. However, the experimental evidence relating smoking to serum BDNF level is controversial with some studies reporting decreased BDNF levels in the serum [34] and plasma [35, 36] of smokers and two studies reporting higher serum BDNF levels in smokers than in non–smokers [22, 37]. Further, a recent report showed that dietay fish oil increased BDNF levels in the rat hippocampus [38]. For this reason we cannot exclude that the admission levels of BDNF could have been influenced by the dietary omega-3 polyunsaturated fatty acids levels in those patients having more than two fish meals per week.

## Conclusions

To our knowledge, a correlation between lipoprotein metabolism and neurotrophins in alcoholic men may provide a deeper insight into the broad field of alcohol-induced changes in neuroactive substances. Because of the expected gender-related differences, further studies are required to test the correlation between neurotrophins and lipids in women with alcohol diseases [32].

## Availability of supporting data

Supporting data is available upon request form the corresponding author.
